# Rapid Improvement of Hyperpigmentation, Growth, and Developmental Milestones With High‐Dose Hydroxocobalamin, Betaine, and Folinic Acid Treatment: The First Patient With Cobalamin G Deficiency in Taiwan

**DOI:** 10.1002/jmd2.70012

**Published:** 2025-04-04

**Authors:** Chi‐Tang Wu, Shih‐Ju Huang, Chu‐Chin Chen, Pao‐Chin Chiu

**Affiliations:** ^1^ Department of Pediatrics Kaohsiung Veterans General Hospital Kaohsiung City Taiwan; ^2^ Division of Pediatrics Pingtung Veterans General Hospital Pingtung County Taiwan; ^3^ Division of Pediatrics Kaohsiung Medical University Gangshan Hospital Kaohsiung City Taiwan

**Keywords:** cobalamin G deficiency, developmental delay, hydroxocobalamin, hyperpigmentation, MTR, whole exome sequencing

## Abstract

In this report, we present the case of a 16‐month‐old patient who was diagnosed with cobalamin G deficiency at 4 months of age via whole exome sequencing by detecting compound heterozygous variants of uncertain significance (VUS) in the *MTR* gene: (1) c.1283T>A, p.Met428Lys; (2) c.2411T>C, p.Ile804Thr. Before the diagnosis, the initial clinical presentation included failure to thrive, skin hyperpigmentation, hypotonia, seizures, and developmental delay. We initiated the treatment with high‐dose parenteral hydroxocobalamin, oral betaine, and folinic acid at 5 months of age (right after receiving WES report). His symptoms, such as skin hyperpigmentation, seizure resolution, developmental delay, and anemia, improved rapidly after the treatment initiation. With treatment, his homocysteine levels declined rapidly and significantly from 117.08 μmol/L at 5 months to 20.23 μmol/L at 5 months and 2 weeks of age. Further, methionine levels increased with treatment from 9.26 μM at 5 months to 14.05 μM at 5 months and 2 weeks of age. The patient is currently receiving intramuscular hydroxocobalamin (2 mg/kg), oral betaine (200 mg/kg), and oral folinic acid (7.5 mg) daily without adverse effects. This case demonstrates the safety and efficacy of early high‐dose parenteral hydroxocobalamin, and oral betaine and folinic acid treatment for cobalamin G deficiency. Moreover, given the patient's clinical manifestations, serologic data, and rapid response to therapy, the *MTR* gene variant previously classified as a VUS should be reclassified as pathogenic and necessitating early diagnosis and treatment.

1

**Summary**

- The clinical effect of high‐dose hydroxocobalamin, oral betaine and folinic acid treatment for CblG. Emphasize the improtance of early diagnosis and treatment. CblG is undetected by newborn screening, and the need for WES. The VUS may indeed pathogenic.

## 2 Introduction

Cobalamin G deficiency (CblG; OMIM #156570) is a metabolic disorder caused by allelic pathogenic variants in the *MTR* gene (5‐methyltetrahydrofolate‐homocysteine methyltransferase). It is characterized by the inability to convert dietary B12 into the cofactor methylcobalamin (the coenzyme for methionine synthase) [1, 2]. In the absence of methylcobalamin, there is an accumulation of homocysteine and decreased synthesis of methionine.

The clinical manifestations of CblG include poor feeding, failure to thrive, hypotonia, seizures, megaloblastic anemia, and neurodevelopmental delays [3, 4]. Its primary treatment is parenteral hydroxocobalamin (OHCbl) and oral betaine. Oral folate/folinic acid and methionine are also recommended [3]. Hydroxocobalamin dosing varies, but published guidelines suggest 0.3 mg/kg/day or 1 mg daily, with dose titration based on the patient's metabolic response [3, 4]. Subsequent dose adjustments may be empirically determined.

Previous literature on CblC (cobalamin C deficiency) suggests that the hydroxocobalamin dosage may be adjusted to address worsening clinical manifestations or to optimize plasma total homocysteine (tHcy), methylmalonic acid (MMA), and methionine [5, 6]. Increased OHCbl doses have been reported to be associated with improved biochemical and clinical outcomes [7]. Moreover, high‐dose prenatal and postnatal hydroxocobalamin treatments improved patients' clinical and biochemical outcomes [8, 9, 10]. However, no published reports or consensus guidelines for dose adjustments in CblG currently exist.

This report presents the clinical and biochemical outcomes of a 16‐month‐old infant who had normal newborn screening results but was diagnosed with CblG at 5 months using whole exome sequencing (WES). High‐dose (2 mg/kg/day) parenteral hydroxocobalamin, oral betaine, and folinic acid treatment were initiated at 5 months of age.

## 3 Case Report

**FIGURE 1:**
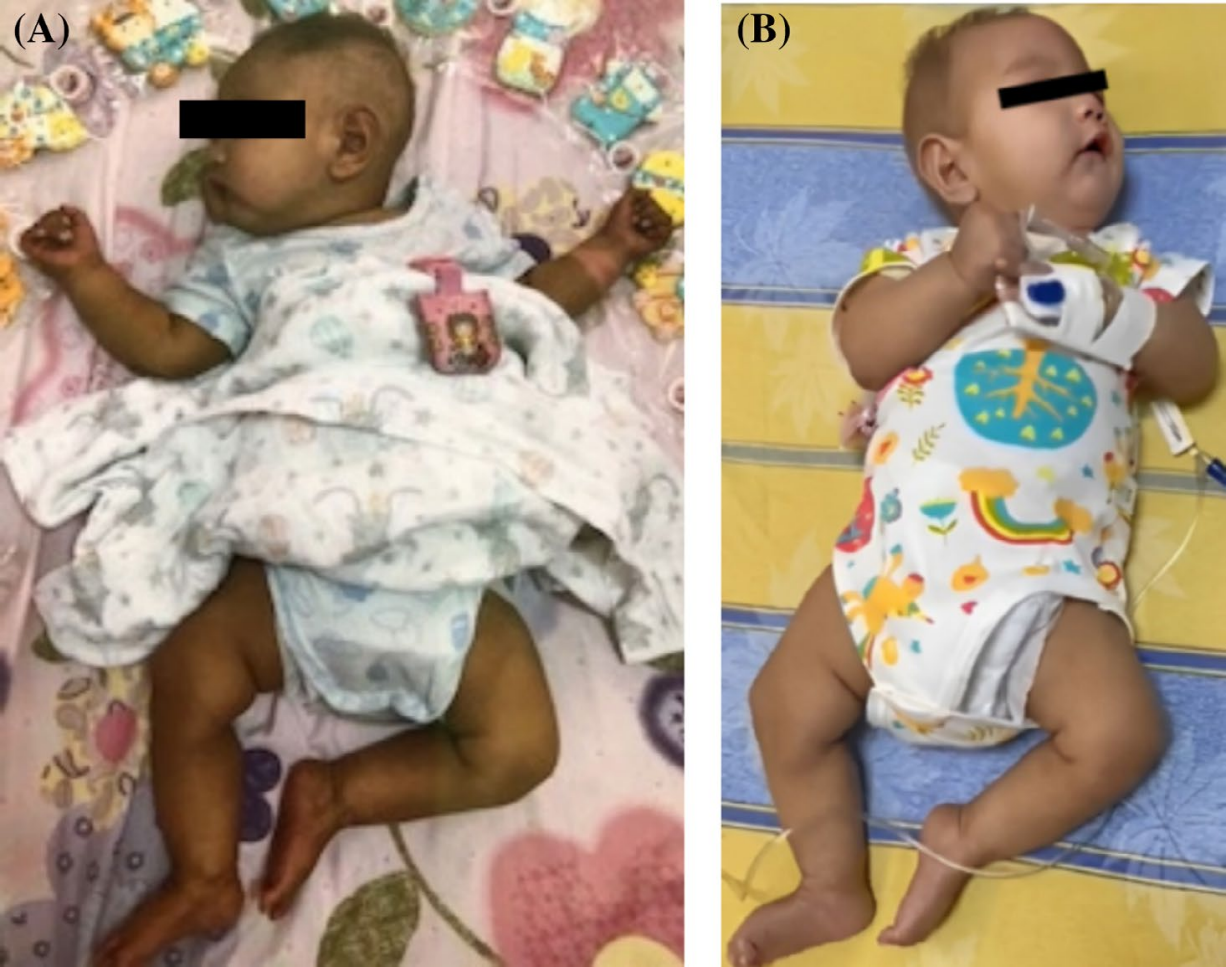
(A) (Left) At 4 months of age, the patient showed skin hyperpigmentation on the neck and four limbs (before treatment). (B) (Right) At 5 months and 2 weeks of age, the patient showed a normal skin color (after a 2‐week high‐dose hydroxocobalamin treatment).

The male patient was born at 37 weeks to nonconsanguineous Han Chinese parents. His birthweight (2526 g) was below the 3rd percentile. The pregnancy and delivery were uncomplicated, and the newborn screening results were normal. Skin hyperpigmentation was first noted on the neck at 2 months of age, which spread to the four limbs at about 4 months of age and then gradually progressed to the whole body (Figure 1A). He was hospitalized at 3 months of age due to high‐pitched crying, hypotonia, seizures, and head lag. His weight (5019 g), height (56 cm), and head circumference (38 cm) were all below the 3rd percentile upon admission. During hospitalization, both electroencephalography (EEG) and brain sonography revealed no abnormality. However, given the persistence of symptoms, he was transferred to the pediatric intensive care unit to undergo 24‐h EEG monitoring and brain magnetic resonance imaging (MRI), which still showed no specific findings. Urine organic acid analysis also revealed no specific findings, prompting the performance of WES at the recommendation of a neurologist and a dermatologist. Two months later, WES revealed compound heterozygous variants of uncertain significance (VUS) in the *MTR* gene: (1) c.1283T>A, p.Met428Lys (not listed in the ClinVar database; all 13 prediction tools indicate that this variant will potentially disrupt the protein structure and function. Classified as VUS according to ACMG interpretation criteria); (2) **c.2411T>C, p.Ile804Thr** (listed in the ClinVar database with a VUS classification) (Variation ID: 962914), 12 of 13 prediction tools indicate that this variant will potentially disrupt the protein structure and function (Classified as VUS according to ACMG interpretation criteria) [11]. CblG was suspected, and the patient was then referred to our pediatric genetic clinic for further confirmation.

**FIGURE 2:**
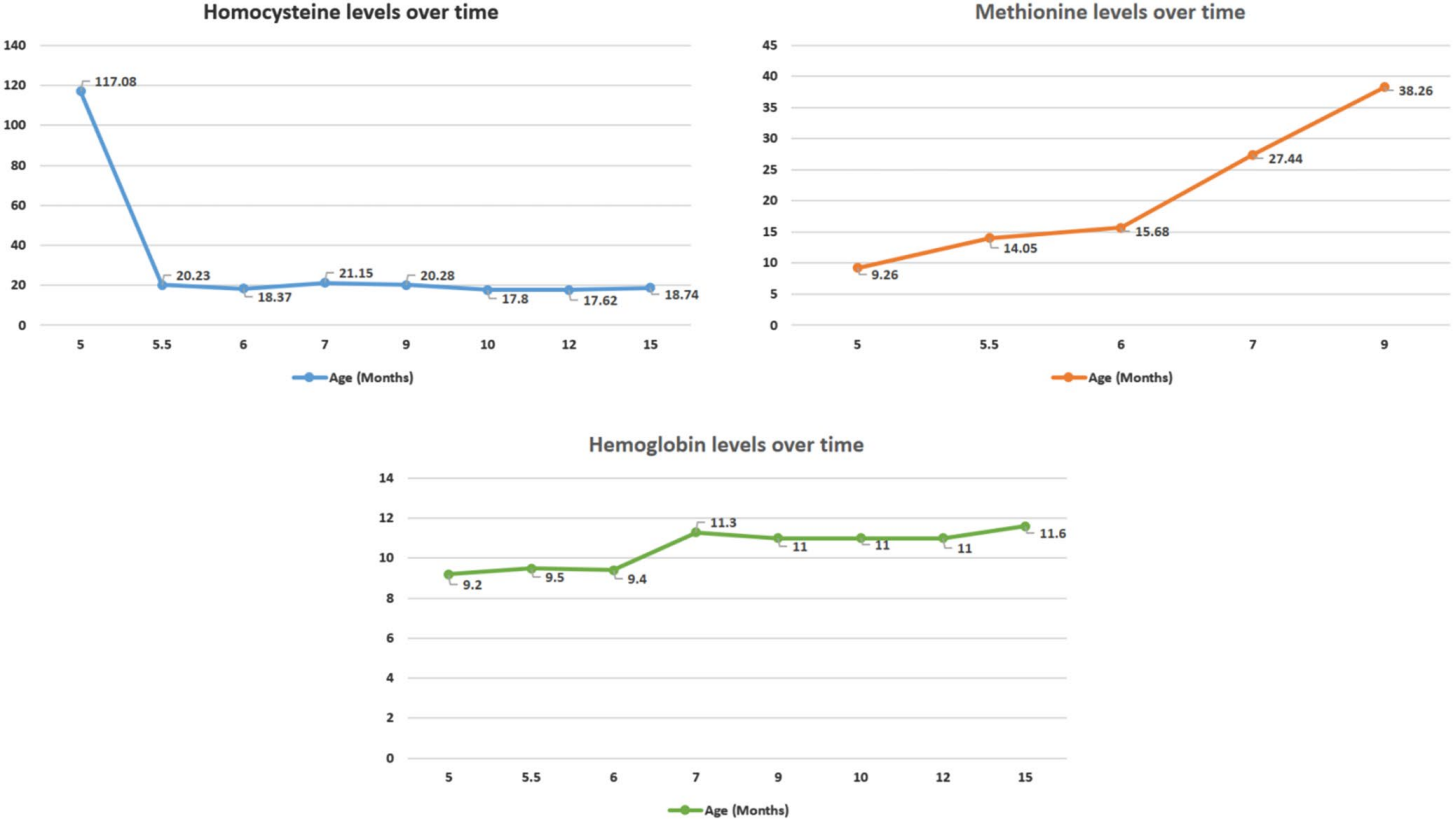
Serum homocysteine, methionine, and hemoglobin levels over time. The normal ranges for homocysteine and methionine are 5–15 μmol/L and 11.93–30.98 μM, respectively.

The serologic data collected at our pediatric genetic clinic included vitamin B12, homocysteine, MMA, and folate levels. Tandem mass spectrometry was also performed. The patient findings were as follows: vitamin B12, 408 pg/mL (reference: 180–914 pg/mL); homocysteine, 117.08 μmol/L (reference: 5–15 μmol/L); MMA, 0.27 μM (reference: 0.01–0.99 μM); and folate, > 24 ng/mL (reference: > 4 ng/mL) (Figure 2). Tandem mass spectrometry showed a decreased methionine level (9.26 μM, reference: 11.93–30.98 μM) (Figure 2), but no increasing C3 carnitine level or increasing C3/C2 ratio. We promptly initiated treatment with 5 mg of parenteral hydroxocobalamin daily (0.87 mg/kg/day) while the patient was 5 months of age. He also received oral betaine (250 mg/kg/day administered in three divided doses) and was placed on an unrestricted diet. Parental Sanger sequencing revealed that both parents carried one variant each of the *MTR* gene (mother: c.1283T>A, p.Met428Lys; father: c.2411T>C, p.Ile804Thr).

One week later, we adjusted the patient's hydroxocobalamin dose to 12.5 mg/day (2 mg/kg/day intramuscular) and oral betaine to 200 mg/kg/day administered in three divided doses. We also added oral folinic acid supplement (5 mg/day, titrated to 7.5 mg/day at 7 months of age) based on a previous study on CblC [9]. The patient's clinical and biochemical parameters, including growth, developmental milestones, neurological status, skin pigmentation, mean corpuscular volume, hemoglobin, homocysteine, and methionine levels, were closely monitored.

**FIGURE 3:**
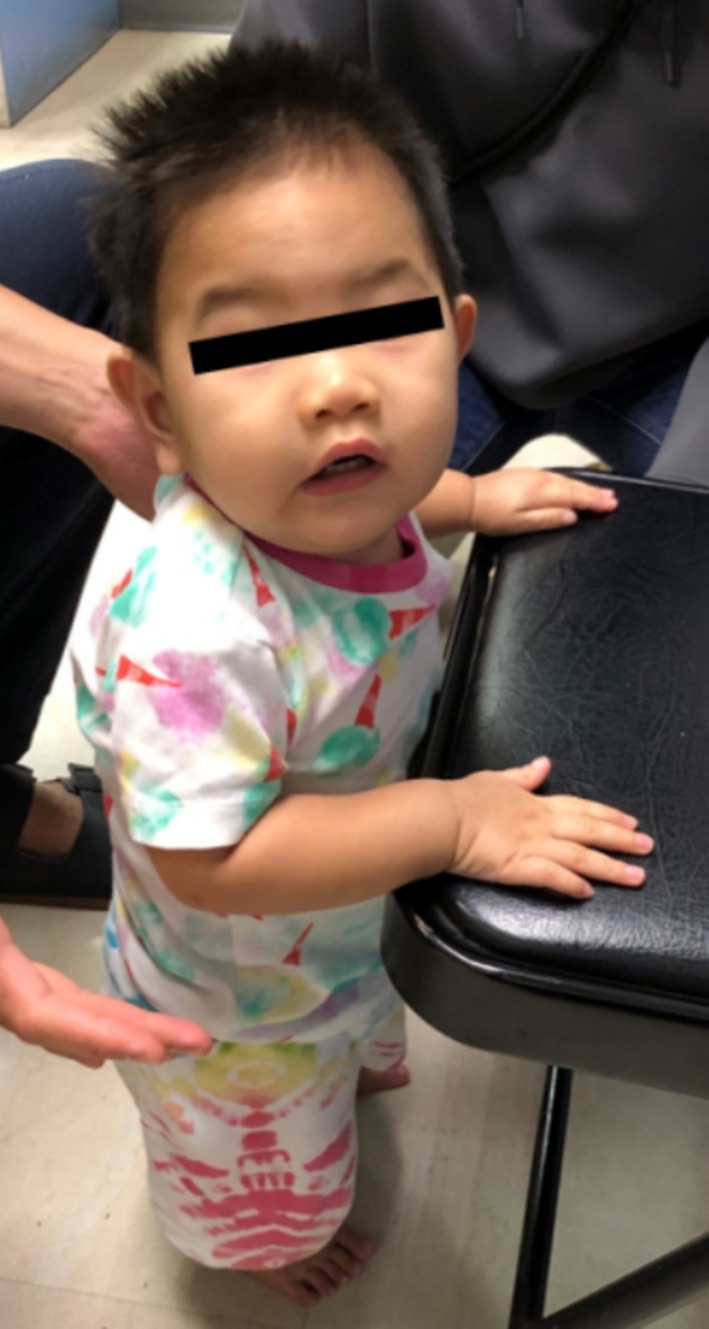
At 10 months of age, the patient showed improvement in growth and achieved the necessary developmental milestones for his age.

Before treatment, the patient exhibited considerable developmental delays, including head lag, absent eye following, inability to hold the head in a prone position, and lack of vocalization at 5 months of age. However, after 5 months of treatment with high‐dose parenteral hydroxocobalamin and oral betaine and folinic acid, he gradually caught up and reached developmental milestones comparable to his age‐matched peers. His skin hyperpigmentation was resolved, and normal skin color returned (Figure 3). Furthermore, the patient's growth parameters improved significantly. His weight, height, and head circumference were 5 kg, 56 cm, and 38 cm, respectively, progressing from all below the 3rd percentile at 4 months of age to 10.5 kg, 78 cm, and 44 cm (15th, 46th, and below the 3rd percentile), respectively, by 16 months of age. His developmental quotient assessment at 14 months of age using the Bayley Scales of Infant Development Version 3 (BSID‐III) revealed the following results: borderline cognitive scale score of 80, equivalent to an 11‐month‐old child; borderline language scale score of 79, with receptive and expressive domains equivalent to those of children aged 11 and 10 months, respectively; delayed motor scale score of 67, with fine and gross motor domains equivalent to those of children aged 9 and 11 months, respectively.

The patient's most recent evaluation at 16 months of age demonstrated significant improvements in developmental milestones (he can clap, take a few steps, and speak some words besides mama/baba), growth parameters, including weight and height, and skin pigmentation. Although microcephaly was still noted, his head circumference still had improvement after treatment (38 cm/0.01 percentile at 4 months of age to 44 cm/0.93 percentile at 16 months of age). Serologic data showed marked improvement in hemoglobin, homocysteine, and methionine levels (Figure 2). No adverse effects of hydroxocobalamin treatment, including erythroderma and chromaturia, were observed [12].

## 4 Discussion

CblG is associated with pathogenic variants in the *MTR* gene [1, 2], which cause abnormal methylcobalamin production and impaired methionine synthase function. The methylation of homocysteine to methionine is then impaired, leading to homocysteine accumulation and decreased methionine synthesis. The typical symptoms include poor feeding, failure to thrive, hypotonia, seizures, megaloblastic anemia, and neurodevelopmental delay.

Before treatment, the patient exhibited the typical symptoms listed above, including failure to thrive, skin hyperpigmentation, hypotonia, seizures, and developmental delay. Serologic data revealed markedly elevated homocysteine and decreased methionine levels. The initial investigations, including newborn screening, EEG, brain sonography, and brain MRI, showed no abnormalities. Subsequent WES revealed compound heterozygous VUS in the *MTR* gene; high‐dose parenteral hydroxocobalamin (2 mg/kg/day), oral betaine, and folinic acid treatment improved the patient's clinical and biochemical outcomes. Based on the clinical manifestations, serologic data, and rapid response to treatment, the initially classified VUS in the *MTR* gene should be reclassified as pathogenic.

The 2017 guidelines for diagnosing and managing cobalamin‐related methylation disorders, including CblG, recommend initiating parenteral hydroxocobalamin treatment at 1 mg/day, with subsequent dose adjustments based on the patient's clinical and biochemical responses [3]. Previous studies on CblC have demonstrated improved biochemical and clinical outcomes with higher hydroxocobalamin doses [5, 6, 7, 8, 9, 10, 13]. Our patient exhibited considerable clinical and biochemical improvements following treatment with high‐dose parenteral hydroxocobalamin. Hydroxocobalamin is generally well tolerated; however, in rare cases, adverse effects, which include erythroderma and chromaturia, have been reported in pediatric patients [11]. Our patient did not experience any adverse effects. Early identification of inborn errors of cobalamin metabolism is crucial.

We initiated treatment immediately after diagnosis, and the patient has since reached normal developmental milestones. This highlights the importance of early diagnosis and treatment in children with such symptoms and/or global developmental delays. Serial biochemical tests and genetic examinations enable early identification of potentially treatable disorders, allowing prompt intervention to correct abnormal values and prevent severe complications before irreversible damage occurs.

Our patient presented with normal skin color at birth, but he developed hyperpigmentation on the neck at 2 months of age, which gradually spread to the entire body. Two weeks of high‐dose parenteral hydroxocobalamin and oral betaine and folinic acid treatment greatly improved the skin pigmentation (Figure 1B). According to recent reports [3, 4, 14], skin manifestations, such as dermatitis, rash, and hyperpigmentation, have been associated with cobalamin metabolism disorders, including CblC, CblF, and CblJ. However, skin hyperpigmentation was not reported in CblG. It is well established that vitamin B12 (cobalamin) deficiency causes skin hyperpigmentation and that the symptoms subside after vitamin B12 supplementation (oral and/or parenteral). However, the pathophysiology of skin hyperpigmentation in CblG remains unclear. Although our patient did not initially have low vitamin B12 levels, skin hyperpigmentation resolved after 2 weeks of vitamin B12 (hydroxocobalamin, intramuscular) supplementation.

The initial newborn screening results were normal. However, CblG was subsequently diagnosed by WES. Unfortunately, CblG was not detected during the newborn screening. According to (1) the 11 routine newborn screenings for diseases outlined by the Health Promotion Administration, Ministry of Health and Welfare of Taiwan (https://www.hpa.gov.tw/Pages/Detail.aspx?nodeid=1139&pid=6577, https://www.hpa.gov.tw/Pages/List.aspx?nodeid=1589) and (2) the diagnosis confirmation guidelines of the Newborn Screening Center, National Taiwan University Hospital (https://www.ntuh.gov.tw/gene‐lab‐nbsc/Fpage.action?muid=3992&fid=3809), homocystinuria is screened if elevated methionine levels are detected. However, the methionine levels decrease in CblG. Newborn screening is also conducted for methylmalonic acidemia and is performed if the C3‐carnitine levels or C3/C2 ratios are elevated. Nevertheless, abnormal C3‐carnitine levels and C3/C2 ratios are not characteristics of CblG. The biomarkers that could detect the other nine disorders during newborn screening are not relevant to CblG. This explains why CblG was not detected during our patient's newborn screening. In two patients with CblG and CblJ from China, newborn screening failed to detect any abnormalities, but they were later diagnosed with the disease through WES [15]. A recent case report also noted that newborn screening programs had low sensitivity, as they primarily rely on methionine and homocysteine levels to detect B12 metabolism disorders. The authors recommended genetic study where necessary [16, 17]. This underscores the importance of maintaining a high index of suspicion for disorders of vitamin B12 metabolism in the differential diagnoses, particularly in patients with suggestive clinical features.

## 5 Summary

Although other remethylation disorders were identified in Taiwan [13], this is the first report of CblG in Taiwan that was treated with high doses of parenteral hydroxocobalamin and oral betaine and folinic acid. The patient initially presented with failure to thrive, skin hyperpigmentation, hypotonia, seizures, and developmental delays, which rapidly improved after early treatment. Further studies are needed to establish the optimal hydroxocobalamin dosing for CblG. However, our report highlights the safety and efficacy of combined treatment with high‐dose parenteral hydroxocobalamin, oral betaine, and folinic acid and the resulting rapid improvements in clinical and biochemical outcomes.

To our knowledge, this is the first documented case of CblG in a Taiwanese patient. The patient exhibited marked clinical and biochemical improvements following high‐dose hydroxocobalamin treatment. This case demonstrates that VUS identified by WES may indeed be pathogenic. A comprehensive evaluation of the patient's clinical presentation and laboratory data is essential for accurate CblG diagnosis with a high suspicion index.

## Author Contributions

Chi‐Tang Wu contributed to drafting a major part of the manuscript, patient management, data interpretation, and critically reviewing and revising the article. Shih‐Ju Huang contributed to patient management and critically reviewed and revised the article. Chu‐Chin Chen contributed to patient management and critically reviewed and revised the article. Pao‐Chin Chiu contributed to patient management, data interpretation, and critically reviewing and revising the article.

## Ethics Statement

All procedures followed were in accordance with the ethical standards of the responsible committee on human experimentation (institutional and national) and with the Declaration of Helsinki, 1975, as revised in 2000. This article does not contain any studies with animal subjects performed by any of the authors.

## Consent

Informed consent was obtained from the caregiver. Additional informed consent was obtained from the caregiver whose identifying information is included in this article.

## Conflicts of Interest

The authors declare no conflicts of interest.

## Data Availability

The authors confirm that the data supporting the findings of this study are available within the article.
